# Educational Trajectories and Academic Achievement from Primary to Secondary Education: A Systematic Review of Individual, Family, School, and Contextual Factors

**DOI:** 10.3390/bs16040608

**Published:** 2026-04-19

**Authors:** Sonia Salvo-Garrido, Karina Polanco-Levicán, Pilar Cisternas-Salcedo, Ana Moraga-Pumarino

**Affiliations:** 1 Departamento de Matemática y Estadística, Universidad de La Frontera, Temuco 4780000, Chile; 2 Escuela de Psicología, Facultad de Ciencias Sociales y Educación, Universidad Santo Tomás, Temuco 4800916, Chile; 3 Independent Researcher, Temuco 4780000, Chile; cisternaspilar@gmail.com; 4 Departamento de Administración y Economía, Universidad de La Frontera, Temuco 4780000, Chile; ana.moraga@ufrontera.cl

**Keywords:** educational trajectories, academic achievement, primary and secondary education, self-regulation, socioemotional adjustment

## Abstract

Educational trajectories developed by students throughout their schooling are shaped by experiences across multiple domains, where learning opportunities coexist with factors that may hinder academic achievement and the development of successful trajectories. The aim of this study was to analyze the personal, family, school, and contextual factors associated with educational trajectories and academic achievement among primary and secondary school students. A systematic review of the literature was conducted based on quantitative longitudinal studies published between 2022 and 2025 and identified through the Web of Science, Scopus, and Education Resources Information Center databases. The results indicate that educational trajectories linked to academic achievement tend to begin in primary education and show relative stability throughout secondary education, with variations over time associated with the interaction of individual, family, school, and contextual factors. These findings have practical implications for behaviorally informed interventions aimed at strengthening self-regulation, teacher support, socioemotional competencies, and family engagement to promote more equitable academic pathways. Overall, the evidence underscores the need to implement comprehensive and differentiated educational interventions articulated across multiple levels to reduce inequalities and foster sustainable academic development.

## 1. Introduction

Educational trajectories associated with the academic achievement of children and adolescents constitute a relevant focus for both educational institutions and scientific research (Sakaki et al., 2024; Wang et al., 2025). These trajectories are progressively shaped throughout the years of schooling and cannot be understood as isolated events; rather, they represent dynamic processes that reflect the interaction of multiple factors influencing learning opportunities and students’ school adjustment (Costa et al., 2024; L. Zhao & Zhao, 2022). In this regard, educational trajectories are influenced by personal, family, school, and contextual factors, which may either foster or constrain academic achievement by directly shaping the conditions under which learning occurs (Costa et al., 2024; Javornik & Klemenčič Mirazchiyski, 2023; L. Zhao & Zhao, 2022).

This process begins early in schooling and may have long-term consequences for students’ future academic opportunities. Evidence indicates that early educational trajectories extend into later stages, influencing students’ persistence within the educational system and their access to more advanced educational opportunities (Costa et al., 2024). Moreover, international comparative studies have shown that academic achievement and educational trajectories are shaped not only by individual characteristics but also by the structural features of educational systems and the inequalities they reproduce (Javornik & Klemenčič Mirazchiyski, 2023). Consequently, it is necessary to adopt an integrated analytical approach to the various factors involved in order to promote more equitable education and to prevent school difficulties in a timely manner.

From a longitudinal perspective, students with higher academic achievement tend to follow upward trajectories, whereas those with lower grades are more likely to remain in declining trajectories over time (Hao et al., 2024). In a similar vein, research examining early childhood development has shown that certain problematic behaviors, such as aggression, are negatively associated with academic achievement; however, this relationship may be moderated by other factors, including the presence of attentional problems (Tamayo Martínez et al., 2021). Likewise, longitudinal studies tracking students from primary through secondary education indicate that children and adolescents with higher levels of resilience exhibit fewer behavioral problems, better socioemotional functioning, and higher academic achievement (Shi et al., 2021).

Accordingly, evidence indicates that different characteristics may operate across educational and developmental stages, influencing academic achievement, although their form or intensity may vary over time (Carpendale et al., 2025). For instance, a meta-analysis reports that motivation toward the attainment of academic goals tends to decline across successive stages of formal education up to university, even when the goals themselves remain relatively stable (Scherrer et al., 2024). In the same direction, orientation toward learning goals decreases progressively over time, particularly during the transition from primary to secondary education (Ramos et al., 2021).

These processes take place within school contexts characterized by specific features, as the quality of educational institutions can support academic achievement through appropriate pedagogical approaches, peer tutoring, the use of educational technologies in instruction, the development of socioemotional competencies, and behavioral interventions aimed at strengthening the school climate (Molloy et al., 2025). In this regard, evidence reported by Kvintova et al. (2025) indicates that achievement outcomes reflect a complex interaction between school adjustment, the development of subject-specific skills—particularly in mathematics and language—and the curricular content addressed within schools.

Notably, a systematic review by Costa et al. (2024), based on 35 empirical studies published between 1930 and 2022, characterizes academic achievement among students aged 10 to 18 years as a phenomenon influenced by multiple factors operating across different domains. At the personal level, salient factors include gender and gender stereotypes, personality traits, cognitive skills, prior achievement, motivation, school-related stress, and substance use. At the contextual level, particularly relevant factors include parental involvement and support, parenting practices, school climate, teaching practices, institutional conditions, and peer relationships.

Scientific literature has emphasized the relevance of multiple levels of influence on educational trajectories, including individual, family, and school-related factors. In addition, the role of local educational policies and their alignment with the sociocultural characteristics of communities has been recognized as central to enhancing the effectiveness of educational interventions (Ferrão & Almeida, 2018). However, despite this recognition, these factors have often been examined in isolation, limiting a comprehensive understanding of how they interact in shaping successful educational trajectories. Consequently, integrated analyses that articulate different levels of influence are essential to inform the design of more effective and equitable educational policies and practices (M.-S. Kim & Park, 2021).

The relevance of the present study lies in its potential to identify personal, family, school, and contextual factors associated with educational trajectories linked to academic achievement among primary and secondary school students, with particular attention to those factors that increase the likelihood of successful schooling processes (Olszewski-Kubilius, 2023). This approach allows for the identification of students whose educational trajectories are characterized by difficulties or lower achievement, thereby supporting the development of targeted school-based interventions. Moreover, it highlights the need to promote more just educational environments for students in contexts of vulnerability, where the educational system plays a fundamental role not only in academic development but also in broader developmental domains (Benner et al., 2021; Burger, 2023; Díaz-Vargas et al., 2023; Dueñas Herrera et al., 2019).

Accordingly, the aim of this study is to analyze the personal, family, school, and contextual factors present in educational trajectories associated with academic achievement among primary and secondary school students.

### Factors Associated with Academic Achievement Trajectories

At the individual level, multiple personal characteristics have been shown to either facilitate or hinder academic achievement in children and adolescents. Academic achievement is consistently associated with intrinsic motivation, which supports students’ engagement and persistence in school-related tasks (Sainz de la Maza et al., 2025; Vergara-Lope Tristán et al., 2017; Santana-Monagas et al., 2025). In addition, students’ self-concept is linked to higher academic expectations and shapes how they respond to academic demands, highlighting the relevance of students’ perceptions of their own performance and competence development for academic achievement (Kalogeropoulos et al., 2023; Elizar, 2021; Zamora-Araya, 2020).

Socioemotional competencies are also associated with academic achievement, particularly intrapersonal skills such as self-awareness and self-management, which show positive and significant associations with reading and arithmetic performance (Martinsone et al., 2025; Y. Zhao & Sang, 2025). These associations are invariant by sex for reading, whereas stronger effects are observed for boys in arithmetic (Carpendale et al., 2025). Academic achievement has also been linked to physical activity through emotional regulation in younger children and behavioral regulation in older children (Vasilopoulos & Ellefson, 2021).

Student engagement is further related to higher grades, more effective use of time and learning environments, and greater behavioral regulation (Estévez et al., 2021), with more engaged students being less likely to have repeated a grade or experienced significant prior learning difficulties (Álvarez-Pérez et al., 2021). Moreover, academic achievement positively predicts self-oriented perfectionism in secondary education (Endleman et al., 2022), whereas fixed mindset beliefs—more prevalent during secondary education—are associated with reduced participation and lower academic achievement (M.-S. Kim & Park, 2021).

Cognitive functioning also plays a central role in academic achievement. Reduced working memory and cognitive flexibility are negatively associated with mathematics performance, while inhibitory control shows a positive association with mathematical ability (Nunes de Santana et al., 2024; Zhu et al., 2025). Attention is critical for language development, with attentional difficulties negatively affecting language-related performance (Santos et al., 2022). Reading comprehension skills contribute positively to achievement in language and literature and to overall academic performance (Molina-Ibarra, 2020; Reina-Reina et al., 2023). Longitudinal evidence further indicates that early linguistic skills predict later mathematics performance (Pazeto et al., 2019). Interconnections among mathematical competencies, linguistic skills, reading comprehension, and creativity have also been shown to support academic achievement (Martínez-Álvarez et al., 2020). Overall, social, cognitive, and emotional development jointly contribute to school performance, with socio-affective competencies being as relevant as cognitive skills for academic success (Hascoët et al., 2020; Santos et al., 2022).

Family-level factors have been consistently associated with students’ academic achievement. Sociodemographic characteristics, such as lower parental educational attainment and lower household income, are negatively related to academic achievement, particularly in mathematics learning (Nunes de Santana et al., 2024). In line with this evidence, research indicates that school-related cultural capital and socioeconomic status—considering parental education and occupation, the number of books at home, and access to goods and services—are relevant for educational attainment and are also linked to academic expectations, internet access, and participation in cultural activities (Vergara-Lope Tristán et al., 2017).

Evidence from studies conducted with Chilean students shows that, alongside the influence of the family’s socioeconomic and educational context on mathematics achievement, parental expectations are also relevant for children’s school performance. These expectations, in turn, are associated with students’ prior academic achievement and with parents’ perceived capacity to support their children’s learning processes (Hascoët et al., 2020).

In addition, parental involvement in school activities has been shown to promote academic success (Balaguer et al., 2020; Jung, 2025). Complementarily, informal family practices related to arithmetic learning and literacy—such as access to books and engagement in educational games—contribute to academic achievement during the early years of schooling (Conica et al., 2023).

At the school level, perceived support from teachers and peers has been shown to foster students’ orientation toward learning goals (Ramos et al., 2021). In this context, teachers and their approaches to mathematics instruction play a central role in student motivation, as linking academic achievement in this subject to long-term goals supports learning processes and school performance (Clements & Sarama, 2025; Ibiapina & Monteiro, 2024). Conversely, grade repetition in primary education is negatively associated with students’ educational trajectories, as those who do not experience repetition tend to show greater persistence, reflected in more favorable long-term outcomes (Ferrão & Almeida, 2018). Accordingly, educational strategies aimed at sustaining student motivation are particularly relevant, given their association with engagement and educational continuity (Scherrer et al., 2024).

## 2. Methods

This study followed the PRISMA (Preferred Reporting Items for Systematic Reviews and Meta-Analyses) guidelines (Page et al., 2021; Rethlefsen et al., 2021). A systematic literature search was performed across multiple databases, including Web of Science (WoS), Scopus, and the Education Resources Information Center (ERIC). The review encompassed a three-year period, including studies published between October 2022 and October 2025. The search and data extraction processes were completed in October 2025.

### 2.1. Search Strategy

The research question guiding this study was as follows: What personal, family, school, and contextual factors are associated with educational trajectories related to students’ academic achievement during primary and secondary education?

The search strategy involved the use of free-text keywords and truncation symbols (*) associated with three main domains: educational trajectories, educational level, and academic achievement, in order to identify longitudinal studies that examined academic and behavioral developmental processes over time. The search string was structured using Boolean operators, combining terms within each domain using OR and linking the three domains using AND operators. Accordingly, the following search string was used: (trajector* OR “Educational trajector*” OR “Learning trajector*” OR “Academic trajector*” OR “Learning progression” OR “Academic development” OR “Educational pathway” OR “Academic path”) AND (“Academic achievement” OR “Academic performance” OR “School achievement” OR “Educational outcomes” OR “Learning outcomes” OR “Grade point average” OR GPA) AND (“Primary school*” OR “Elementary school*” OR “Early grade” OR “Basic education” OR “formal education” OR “primary education” OR “elementary education” OR “secondary education” OR “secondary school*” OR “high school*” OR “middle school*” OR K-12).

Searches were conducted within database-specific metadata fields, including Topic (TS) in Web of Science, TITLE-ABS-KEY in Scopus, and All Text (TX) in ERIC (EBSCOhost). Although field tags differ across databases, all searches were restricted to comparable metadata fields (titles, abstracts, keywords, and descriptors), ensuring conceptual consistency across platforms. The strategy was designed to identify longitudinal studies examining educational trajectories over time, including the complete Boolean syntax, field specifications, and applied database filters, which are described in the present section.

The search strategy focused on identifying studies examining educational trajectories and academic achievement, while associated factors were identified during the screening and data extraction phases.

### 2.2. Inclusion and Exclusion Criteria

#### 2.2.1. Inclusion Criteria

Articles eligible for this study examined educational trajectories across both primary and secondary education among children and adolescents, typically ranging from approximately 6 to 18 years of age. Only studies that included data from both educational stages within the same study were considered eligible. In studies involving broader age ranges, only data corresponding to these educational levels were included in the analysis, while information related to earlier or later stages was excluded. In addition, selected studies were required to report empirical findings on factors associated with educational trajectories related to academic achievement. Academic achievement was considered the primary outcome of interest. No specific predictors were predefined; instead, studies were included if they reported associations between empirically examined factors and trajectories of academic achievement over time.

Only quantitative longitudinal studies published as peer-reviewed journal articles and written in Spanish or English within the period from October 2022 to October 2025 were included.

#### 2.2.2. Exclusion Criteria

Studies that examined only one educational stage (either primary or secondary education) without addressing both were excluded. Research focused exclusively on preschool populations, university students, or teachers was also excluded. With respect to publication type, systematic reviews, book chapters, conference proceedings, letters, and book reviews were not considered. Finally, studies published in languages other than Spanish or English were excluded.

### 2.3. Participants

Children and adolescents were included if they participated in studies that considered at least two educational stages: primary and secondary education. Given that the age of entry into primary education may vary across countries where the studies were conducted, reference was made to UNICEF (2022), which indicates that primary education is typically designed for children aged 6 to 11 years. Nevertheless, this range may be more flexible, extending from approximately 6–7 to 12–13 years, although primary education is generally completed at around 12 years of age (OECD/Eurostat/UNESCO Institute for Statistics, 2015). This stage has an approximate duration of six years. Subsequently, secondary education—comprising lower secondary and upper secondary education—begins and has an approximate duration of an additional six years (OECD/Eurostat/UNESCO Institute for Statistics, 2015).

### 2.4. Study Selection

The identified records were imported into the web- and mobile-based application Rayyan (Ouzzani et al., 2016). In parallel, spreadsheets were used to record the number of studies, reasons for exclusion, and the different stages of the selection process. The initial screening was conducted based on the titles and abstracts of the articles, allowing for the identification of those that met the predefined inclusion and exclusion criteria. Rayyan facilitated the identification and removal of duplicate records through both automated and manual procedures.

The screening process was conducted independently by at least two reviewers, with disagreements resolved through discussion. Inter-rater reliability was not formally calculated, as disagreements were minimal and resolved through consensus.

For the full-text review, the selected articles were distributed equally among the researchers. In cases where discrepancies regarding the inclusion of a study arose, these were discussed jointly until consensus was reached. Subsequently, the systematic extraction of relevant information was initiated, along with the development of a summary table.

### 2.5. Data Extraction and Thematic Analysis

A table was developed to compile the information extracted from the selected articles. The table included (1) study information (authors and year of publication, country, and study design); (2) participants (sample size and characteristics, including age, grade, and gender); (3) the study objective; (4) key background variables examined; (5) the main domain of association; (6) the main results; and (7) the direction of association identified. Studies were also coded according to the ecological level of predictors examined (individual, family, school, and contextual or structural domains). When studies included predictors from multiple ecological levels, the main domain of association was determined based on the primary construct analyzed in relation to academic achievement trajectories. Because several studies examined variables from more than one domain, domain frequencies reflect multi-domain coding rather than mutually exclusive classification. This information is presented in Table S1.

The analysis of the findings from each selected article was conducted in accordance with the guidelines for thematic analysis (Braun & Clarke, 2006; Clarke & Braun, 2017), following an inductive approach that allowed categories to emerge from the data, resulting in the identification of thematic categories.

The process began with a detailed reading of the articles, which enabled the identification of emerging categories. Subsequently, the resulting categories were compared, and similar categories were synthesized. This stage involved the entire research team, facilitating the comparison of perspectives, the refinement of categories, and consensus regarding the results.

No meta-analysis was performed due to substantial heterogeneity across studies in research designs, measurement instruments, outcome indicators, and trajectory modeling approaches, which precluded a meaningful quantitative synthesis.

### 2.6. Risk of Bias Assessment

The risk of bias was assessed using the Joanna Briggs Institute (Joanna Briggs Institute, 2020) critical appraisal tool for longitudinal observational studies. This instrument provides a standardized, design-specific framework for evaluating methodological quality, including participant recruitment, measurement of variables, control of confounding factors, and the reliability and validity of outcome measures. The assessment was conducted independently by two reviewers, and any discrepancies were resolved through discussion until consensus was reached; when necessary, a third reviewer was consulted to reach a final decision (Porritt et al., 2014).

Each study was evaluated across the criteria included in the JBI checklist, with responses categorized as “Yes”, “No”, “Unclear”, or “Not applicable”. As a result of this process, one study (Morosanova et al., 2023) was excluded due to a high risk of bias, as it did not report psychometric indicators that would allow for the assessment of the reliability and validity of the instruments used, despite having a robust longitudinal design.

In total, 45 studies were classified as high quality, 9 as moderate quality, and 1 as low quality. Studies with ratings including “Unclear” responses in specific JBI items (Bouffard et al., 2023; Carroll et al., 2022; S. Y. Kim et al., 2025; Mädamürk & Kikas, 2024; Oh, 2023; Quintero & Wang, 2023; Wang & Tang, 2024; Zhang et al., 2024; J. Zhou & Hawrot, 2025), although achieving high overall scores, presented limitations related to specific psychometric properties. These included low or unreported reliability coefficients and the use of subjective or non-standardized measures of academic performance, introducing a potential risk of information bias. Nevertheless, as these studies implemented advanced analytical strategies for controlling confounding variables and longitudinal modeling, their inclusion was considered to contribute substantively to the synthesis of the available evidence.

## 3. Results

**Figure 1:**
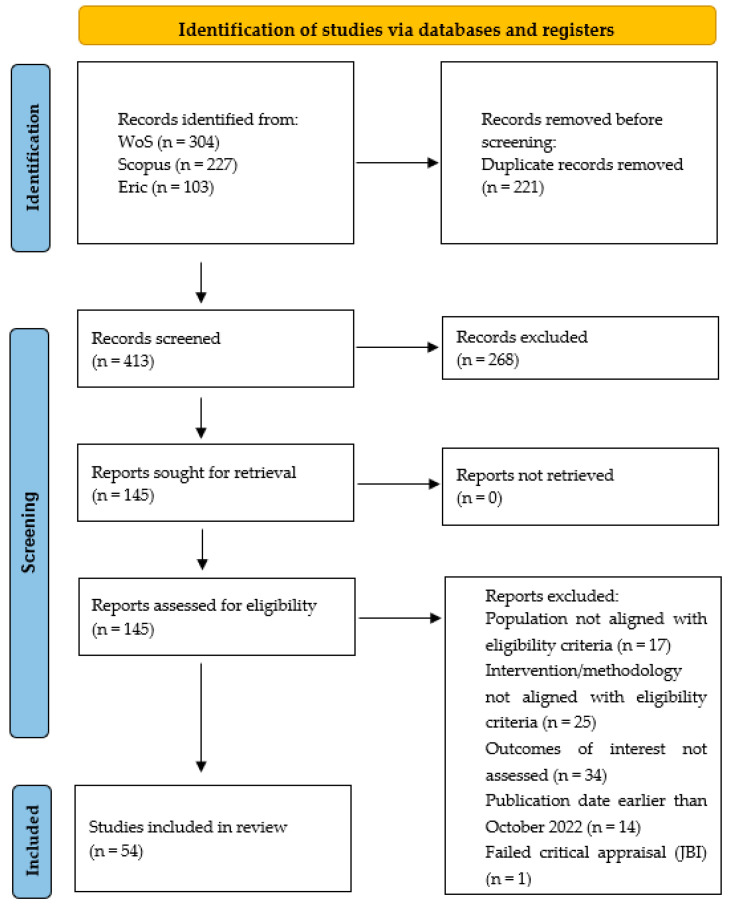
PRISMA flow diagram.

The systematic search initially identified a total of 634 records across three major databases (WoS, n = 304; Scopus, n = 227; ERIC, n = 103). After the removal of 221 duplicate records, the titles and abstracts of 413 articles were screened according to the predefined eligibility criteria, resulting in the preliminary exclusion of 268 records that did not meet the established inclusion criteria. Subsequently, 145 articles were retrieved and assessed for full-text eligibility. During this phase, 90 reports were excluded based on specific predefined criteria: population characteristics not aligned with the study focus (n = 17), intervention or methodological design not meeting eligibility requirements (n = 25), outcomes of interest not assessed (n = 34), and publication date prior to October 2022 (n = 14). Of the 55 studies that met all inclusion parameters, one additional study was excluded following the JBI critical appraisal due to a high risk of methodological bias. Consequently, a final sample of 54 studies was retained for data extraction and synthesis. The study selection process is illustrated in the PRISMA 2020 flow diagram (Figure 1).

### 3.1. Factors Associated with Educational Trajectories and Academic Achievement

In order to organize the evidence derived from the included studies, four categories of factors associated with students’ educational trajectories and academic achievement were identified: individual, school, family, and contextual factors. These categories emerged inductively from the findings reported in the reviewed studies.

#### 3.1.1. Individual-Level Characteristics Influencing Educational Trajectories Associated with Academic Achievement

Across the reviewed studies, individual-level factors were grouped into relatively consistent patterns linking student characteristics to differentiated academic performance trajectories. Overall, the evidence indicates that trajectories of academic achievement are associated with (a) gender-related differences; (b) socioemotional competencies and psychosocial adjustment; (c) self-regulation, motivation, and academic goals; (d) cognitive and metacognitive skills; and (e) conditions of vulnerability or special educational needs (Table S1).

#### 3.1.2. Gender and Academic Trajectories

Studies report gender differences in academic performance over time, with more favorable trajectories for females in certain academic domains, such as reading comprehension (Erentaitė et al., 2023), as well as higher initial levels and greater growth rates in second language achievement (Oh, 2023). These patterns, however, coexist with less favorable indicators of emotional adjustment, as being female has been associated with higher levels of test anxiety during school transitions (Fréchette-Simard et al., 2023).

#### 3.1.3. Socioemotional Competencies, Peer Relationships, and Psychosocial Adjustment

The evidence indicates that socioemotional behaviors and resources are consistently associated with more favorable academic trajectories. For example, students exhibiting higher levels of prosocial behavior also demonstrate higher academic achievement, whereas lower prosociality and reduced assertiveness are associated with poorer academic outcomes (Ma et al., 2026).

At the relational level, studies conducted with Chinese students showed that greater popularity was associated with higher academic performance and that increases in popularity predicted subsequent improvements in academic achievement. In contrast, peer acceptance did not demonstrate longitudinal effects on academic performance (Wei et al., 2023). Importantly, academic performance is not exclusively associated with positive factors. In the study by Beattie et al. (2025), adolescents who exhibited chronically elevated levels of loneliness showed a slightly higher grade point average (GPA) compared to other groups.

Additionally, lower initial performance in mathematics was associated with higher levels of test anxiety at the end of primary education; however, academic performance did not predict changes in anxiety during the transition to secondary education (Fréchette-Simard et al., 2023). Conversely, higher academic performance was associated with a lower likelihood of belonging to a trajectory characterized by initially elevated levels of internet addiction and depressive symptoms that increase jointly over time (Zhang et al., 2024).

#### 3.1.4. Self-Regulation, Motivation, and Performance Stability

Several studies converge in showing that self-regulation and motivation underpin differentiated academic trajectories. Self-regulation linked to recognizing the personal value of studying is positively associated with higher prior academic performance (Alivernini et al., 2023), suggesting that early academic success may buffer subsequent declines in autonomous motivation over time. Consistent with this pattern, academic performance trajectories tend to exhibit longitudinal stability, with prior performance exerting a strong influence on later outcomes (Johnson et al., 2022).

Self-regulated learning, entailing the use of planning, monitoring, and goal-setting strategies as well as the capacity to sustain motivation, was positively associated with higher performance on standardized assessments. By contrast, externally regulated learning showed no significant associations with academic outcomes (Bardach et al., 2023). Along similar lines, satisfaction of basic psychological needs (autonomy and competence) significantly predicted initial levels of self-regulated learning; moreover, the need for relatedness with peers demonstrated a significant effect only within the high-achievement group (Lee et al., 2025), indicating differential mechanisms according to achievement level.

#### 3.1.5. Goals, Expectations, and Educational Aspirations

As noted above, motivation represents a relevant variable in understanding students’ educational trajectories and academic performance. Student profiles characterized by high-quality motivation (i.e., high autonomous motivation) show a greater likelihood of belonging to favorable mathematics performance trajectories, whereas low-quality motivation is associated with low-growth or declining trajectories (Lyu & Hu, 2024). During middle school, achievement goals follow a developmental pattern marked by a decline in task-oriented goals, indicating a reduced orientation toward learning, personal improvement, and self-improvement goals (Peixoto et al., 2025).

More specifically, outcome goals remain stable and predict increases in academic grades, whereas work-avoidance goals are associated with lower GPA and higher levels of school burnout, both at baseline and longitudinally (Yu et al., 2023). In addition, initial educational expectations predicted growth in science performance during secondary education and, subsequently, a higher likelihood of obtaining credentials in STEM fields (Yeung & Igarashi, 2025). Educational aspirations, both idealistic and realistic, were also positively associated with academic performance, with this relationship strengthening over time (Becker et al., 2023). Finally, certain complex motivational profiles, such as struggling ambitious, exhibit high performance in mathematics and literacy despite high perceived costs, even outperforming profiles characterized by positive engagement (Raufelder et al., 2022).

#### 3.1.6. Emotional–Behavioral Dysregulation and Temperament

The co-occurrence of emotional, behavioral, and attentional problems constitutes a dysregulation profile associated with lower initial scores and slower growth rates in language and mathematics across primary and secondary education; however, this profile does not necessarily determine membership in a low-achievement trajectory (Shi et al., 2023). In addition, greater sensitivity to negative stimuli (Behavioral Inhibition System; BIS) and a strong reward orientation (Drive/Behavioral Activation System; BAS) were associated with a higher likelihood of belonging to low and declining academic performance trajectories at the end of primary education and the beginning of lower secondary education (Høstmælingen et al., 2025).

#### 3.1.7. Cognitive Skills and Metacognition

The evidence indicates that academic performance trajectories are associated with the use of metacognitive strategies, such that students who employ these strategies from early stages also report a higher valuation of education as a means for goal attainment (Cai et al., 2023). Additionally, competencies such as calculation and word-problem-solving skills are associated with sustained superior performance and higher scores on subsequent mathematics and language assessments (Mädamürk & Kikas, 2024).

In this context, perceived problems in working memory predicted higher levels of negative teacher–student interactions, whereas greater closeness in the teacher–student relationship was associated with subsequent reductions in these perceived difficulties. Notably, a bidirectional relationship exists between perceived working memory problems and arithmetic performance across the educational trajectory (Sankalaite et al., 2023).

Within the broader framework of cognitive and metacognitive functioning, effortful control—understood as the self-regulatory capacity to inhibit impulsive responses and direct attention toward goals—has been identified as a key predictor of long-term academic performance (Cheng et al., 2024). Likewise, Qi et al. (2025) examined students’ beliefs about the nature of intelligence (intelligence mindsets), identifying differentiated longitudinal profiles: a growth mindset, referring to the belief in the possibility of developing intelligence and abilities through effort and learning, and a fixed mindset, which reflects the belief that intelligence tends to be stable. The results showed that the growth profile (high growth mindset and low fixed mindset) exhibited the highest levels of academic performance over time (Qi et al., 2025).

#### 3.1.8. Special Educational Needs and Vulnerability Trajectories

Students with special educational needs exhibit lower performance in reading and mathematics compared to their peers, with these gaps remaining stable over time (Carroll et al., 2022; Erentaitė et al., 2023). Among students with ADHD, consistently lower grade trajectories have been observed, with effects that are more pronounced at the beginning of secondary education and gradually attenuate toward its end (Lange-Küttner et al., 2025). Nevertheless, adolescents with ADHD continue to demonstrate lower performance on standardized assessments throughout secondary education, with important implications for access to higher education (Di Lonardo Burr et al., 2022). Finally, with regard to culturally situated psychological resources, students exhibiting high or increasing trajectories of Psychological Suzhi demonstrated superior and sustained academic performance (Nie et al., 2025), whereas early psychosocial risk was associated with significantly lower performance from 3rd to 8th grade (Farley et al., 2023).

### 3.2. School-Level Characteristics Influencing Educational Trajectories Associated with Academic Achievement

At the school level, the findings converge in showing that academic performance trajectories are shaped by relational, motivational, and contextual mechanisms that operate consistently over time. In particular, the evidence highlights the role of perceived teacher support and the quality of the teacher–student relationship as central determinants of student engagement and behavioral adjustment, which, in turn, are associated with differentiated trajectories of academic achievement.

Perceived teacher support—including emotional dimensions (advocacy), instructional dimensions (expectations, organization and clarity, feedback–feedforward, and instructional relevance), and classroom management—was positively associated with student motivation (valuing, growth goals, and aspirations). These motivational resources, in turn, significantly predicted student engagement and academic performance in language and mathematics (Martin et al., 2024). Along similar lines, the quality of the teacher–student relationship showed longitudinal associations with indicators of academic and behavioral adjustment: higher levels of conflict were related to lower academic performance, whereas teacher–student closeness was associated with higher academic achievement (Magro et al., 2025).

Beyond relational dynamics, instructional orientations within the classroom also appear to play a relevant role. More specifically, perceptions of classroom structures oriented toward mastery goals, defined as instructional practices that emphasize learning, personal progress, and improvement, were associated with greater growth in academic achievement among low-performing students, regardless of socioeconomic status (Quintero & Wang, 2023).

The effects of instructional practices, however, were not uniform across developmental stages. The findings indicate that certain instructional dimensions vary according to students’ level of maturity. For example, classroom management was positively associated with academic grades; by contrast, in primary education, excessive goal clarity was negatively related to student motivation. This association became positive toward the end of primary education and the beginning of secondary education, reflecting developmental changes in how students process and respond to school demands (J. Zhou & Hawrot, 2025).

Some studies have examined the relationship between the ethnic or racial composition of the school environment and students’ academic achievement. In this regard, the evidence indicates that teachers’ racial or ethnic background may be associated with academic gains for specific groups of students. For example, Black students taught by teachers of the same race were found to show improvements in mathematics and English Language Arts (ELA) during primary education, particularly in self-contained classrooms; however, this pattern was not observed in secondary education (Hwang et al., 2023).

Ramlackhan and Wang (2024) examined achievement trajectories in mathematics and ELA across urban school districts in the United States and found that these trajectories were linked to structural characteristics of the school context, including district racial composition and the proportion of students in poverty. Specifically, districts with a higher proportion of Hispanic students tended to be associated with less favorable mathematics achievement trajectories, whereas those with a higher proportion of White students tended to be associated with more favorable trajectories. Similarly, higher levels of socioeconomic disadvantage, operationalized, for example, as the proportion of students receiving free lunch, were related to lower academic trajectories.

Furthermore, higher initial levels of classroom poverty were associated with significant reductions in test scores and a lower likelihood of high school completion and college enrollment. These differentiated effects were reported to disproportionately affect students from low- and middle-income families and were associated with reduced student motivation, deterioration of classroom climate, lower parental involvement, and decreased teacher attention (Huang et al., 2025).

With respect to the temporal dynamics of academic trajectories, the evidence indicates that prior achievement is a robust predictor of later performance, with middle school scores significantly predicting outcomes in high school (Johnson et al., 2022). Additionally, trajectories of mathematical growth between 4th and 6th grade were associated with mathematics achievement in 8th grade, with initial performance levels playing a decisive role (Yeo et al., 2023). Within the same study, direct instruction was linked to higher achievement among students following high and average trajectories, whereas dialogic instruction was associated with better outcomes among students in low-achievement trajectories, suggesting that the effectiveness of pedagogical practices may vary according to students’ longitudinal performance profiles (Yeo et al., 2023).

A comparable pattern of stability was also observed in English achievement among South Korean students. Although students with lower initial performance improved over time, they did not reach the achievement levels of their higher-performing peers. This persistent gap was associated with early exposure to private tutoring, particularly in urban contexts (Ryu & Lee, 2024).

In parallel, research addressing contexts of human mobility indicates that migrant students often exhibit lower initial academic performance due to limited exposure to the language of instruction and the local curriculum. Nevertheless, they demonstrate significant progress over time, with normative adaptation not substantially altering this pattern (Le Pichon et al., 2023). Consistent with these findings, language support programs were associated with positive and statistically significant improvements in mean English Language Arts (ELA) scores among English learners, although their performance remained below that of students with higher language proficiency (Ochoa et al., 2024).

Other school-level findings point to complex associations between average school achievement and student well-being. While higher average school performance was linked to the development of subjective well-being during secondary education, higher-achieving school contexts were also associated with greater declines in individual life satisfaction. This pattern suggests that environments characterized by high-performing peers may negatively influence students’ self-perceptions (Wu & Becker, 2023). In addition, trajectories of formal school exclusion showed differential associations with educational attainment, such that students experiencing a higher number of formal exclusions displayed a lower likelihood of obtaining General Certificate of Secondary Education (GCSE) qualifications (Tseliou et al., 2024).

Finally, several studies identified positive effects associated with specific interventions or educational modalities. Full-time schools were related to more favorable academic trajectories by increasing the likelihood of attending more selective secondary schools and attaining higher scores on admission examinations, reflecting cumulative long-term effects (Cabrera-Hernández et al., 2023). In addition, moderate engagement in structured extracurricular activities was linked to improvements in mathematics and reading achievement, whereas participation in unstructured activities showed no significant relationship with academic performance (Connelly et al., 2024).

### 3.3. Family-Level Characteristics Influencing Educational Trajectories Linked to Academic Achievement

The evidence indicates that trajectories of academic achievement are shaped by both structural conditions (e.g., parental educational level, income, and residential context) and relational and socialization processes (parenting practices, expectations, and parental involvement). Together, these factors configure cumulative pathways of risk and protection that manifest longitudinally in students’ academic performance.

In structural terms, low parental educational attainment, limited family income, and neighborhood of residence are associated with trajectories of lower academic achievement, which are in turn linked to the accumulation of early psychosocial risks, particularly in contexts of socioeconomic vulnerability (Carbonneau et al., 2023; Larsen et al., 2023). Parental educational level is positively associated with achievement in mathematics and physics (Y. Zhou & Lian, 2024), while socioeconomic status shows a positive relationship with mathematics achievement, with better academic outcomes observed among students from families with higher parental educational levels and higher household monthly income (Yeo et al., 2023).

Regarding parenting processes, maladaptive parenting styles are associated with an increased likelihood of belonging to risk trajectories in which academic difficulties and mental health problems co-occur. Specifically, severe punitive control is associated with a higher probability of belonging to the group characterized by high levels of psychopathological symptoms and low academic achievement. In contrast, psychological control is observed among students with medium to high academic performance but elevated levels of internalizing and externalizing symptoms, indicating that academic performance does not necessarily reflect favorable psychosocial adjustment (Li et al., 2025).

Consistently, parental educational expectations are positively associated with both the initial level and the growth rate of mathematics achievement across adolescence, regardless of gender (Wang & Tang, 2024). By contrast, forms of parental involvement at home based on control and supervision (e.g., homework monitoring or screen-time control) show more limited associations with academic achievement, with significant relationships observed primarily for initial performance (Wang & Tang, 2024). The perception of parental support not conditioned on academic performance is associated with better academic achievement, although of marginal magnitude, as well as with better emotional functioning, higher motivation to learn, greater self-regulation, better time management, and less favorable attitudes toward school dropout, compared to students who perceive parental affection as more strongly conditioned on academic performance (Bouffard et al., 2023).

Finally, within migrant populations, specific family mechanisms associated with academic achievement trajectories have been identified. Among Chinese American adolescents, bicultural orientations—particularly more U.S.-oriented maternal orientations—were associated with increases in GPA through lower acculturative stress, higher levels of positive parenting behaviors, and reduced mother–adolescent alienation (Lo et al., 2025). For adolescents of Mexican origin, perceived academic discrimination was associated with lower secondary school grades and reduced educational expectations through indirect mechanisms; this association was further moderated by language brokering processes, such that more positive parent–child relationships attenuated the negative association between academic discrimination and academic achievement (S. Y. Kim et al., 2025).

### 3.4. Contextual-Level Characteristics Influencing Educational Trajectories Linked to Academic Achievement

At the contextual level, the results indicate that macrosocial variables and collective events can exert sustained effects on academic achievement trajectories, particularly among students in situations of socioeconomic vulnerability. Specifically, students with lower socioeconomic status show a higher probability of belonging to less favorable trajectories in mathematics (Erentaitė et al., 2023).

In addition, the COVID-19 pandemic emerged as an unexpected contextual event with longitudinal effects on academic performance, particularly in reading. Among students in grades 3 through 8, a significant decline in reading test scores was observed relative to the pre-pandemic period (2019), with larger decreases in lower grades (grades 3 to 5), indicating a cumulative and progressive pattern over time (Kuhfeld et al., 2023). However, the evidence is not entirely homogeneous: in a Swiss study, an eight-week school closure was not associated with significant losses in mathematical competencies, and longitudinal increases were reported across the 2019–2020 school year (Compagnoni et al., 2025).

### 3.5. Integrative Synthesis of the Results

The findings of this systematic review indicate that educational trajectories associated with academic achievement are organized into relatively stable longitudinal patterns that vary according to individual, family, school, and contextual characteristics. Across the reviewed studies, these trajectories are described as cumulative processes in which academic achievement is systematically associated with indicators of behavioral, socioemotional, and cognitive adjustment over time.

At the individual level, the results indicate that self-regulation, autonomous motivation, adaptive academic goals, and cognitive and metacognitive skills are consistently associated with more favorable academic achievement trajectories. In parallel, the presence of emotional, behavioral, or attentional difficulties is related to lower initial performance levels and slower growth rates. Nevertheless, the reviewed studies show intraindividual variability and do not support a uniform determination of persistently low achievement trajectories. In addition, a high degree of longitudinal stability in academic achievement is observed, with a persistent association between prior performance and subsequent outcomes.

At the school level, the findings converge in showing that perceived teacher support, the quality of the teacher–student relationship, and mastery-oriented pedagogical practices are associated with systematic differences in academic achievement trajectories. However, these associations vary according to educational level, initial performance profile, and the socioeconomic context of the school, resulting in differentiated patterns of growth and maintenance of achievement across schooling.

With regard to the family context, the results show that academic trajectories are linked to a combination of structural and relational factors. Parental educational level and economic resources are consistently associated with academic achievement, while processes such as educational expectations, parenting styles, and parental support are related to differences in the longitudinal evolution of performance. In particular, the studies indicate that parental support not conditioned on academic achievement is associated with trajectories characterized by greater stability and more favorable emotional and motivational adjustment.

Finally, the results indicate that broader contextual factors are associated with variation in academic achievement trajectories. Socioeconomic inequality and exposure to disruptive events are linked to significant changes in achievement patterns, with cumulative and unequal effects across student groups. Evidence related to the COVID-19 pandemic shows heterogeneous declines and recoveries across countries, educational levels, and academic domains.

In quantitative terms, the distribution of predictors across the reviewed literature reveals a clear predominance of individual-level explanatory frameworks. Of the 54 included studies, 87.0% examined individual-level predictors, 68.5% incorporated school-level factors, 51.9% considered family-level variables, and 27.8% explicitly addressed broader contextual or structural determinants. Because many studies included predictors from more than one ecological domain, these percentages are not mutually exclusive. This pattern suggests that although academic achievement trajectories are conceptualized within multilevel ecological frameworks, empirical research continues to emphasize intra-individual processes more strongly than systemic or structural conditions shaping long-term educational development.

Across the included studies, academic achievement trajectories are characterized by the coexistence of stability and change and by systematic associations with individual, family, school, and contextual factors over time.

## 4. Discussion

The analysis of educational trajectories associated with academic achievement during primary and secondary education is essential for understanding persistence within the educational system and subsequent academic outcomes. The results of this review indicate that such trajectories are not driven by a single factor but instead emerge from longitudinal interactions among individual, family, school, and contextual variables, in line with previous evidence (Costa et al., 2024). From a behavioral and developmental perspective, academic achievement can be understood as a complex indicator of school adjustment shaped over time by processes of self-regulation, motivation, socialization, and contextual influences.

The heterogeneity of educational systems across countries represents an important limitation for the interpretation of the findings. Differences in institutional structures, curricular frameworks, and educational policies may influence the nature and strength of the associations observed across studies. Consequently, the comparability of results across contexts is constrained, and the findings should be interpreted with caution. Rather than supporting universal generalizations, this review aims to identify recurring patterns and associations that emerge across diverse educational settings.

From a quantitative perspective, the review also reveals a marked imbalance in the empirical focus of the field. Although academic achievement trajectories are theoretically framed within multilevel ecological models, the majority of studies primarily emphasize individual-level predictors, while comparatively fewer incorporate structural or contextual determinants. This distribution suggests that, despite the recognition of multilevel influences, research continues to privilege intra-individual explanatory mechanisms over broader systemic conditions that shape long-term educational development, with contextual factors being less frequently incorporated in empirical studies on educational trajectories.

It is important to acknowledge that the search strategy adopted in this review may have limited the identification of certain contextual and structural factors, such as migration-related processes, school leadership, and broader systemic characteristics of educational systems. As a result, these dimensions may be underrepresented in the reviewed literature. This pattern likely reflects both prevailing trends in empirical research—where individual-level variables are more frequently examined—and, to some extent, the scope of the search strategy employed. Consequently, the findings should be interpreted with caution, particularly regarding the relative weight assigned to contextual factors. Future research would benefit from more targeted search strategies and integrative approaches that explicitly incorporate structural and contextual dimensions to achieve a more comprehensive understanding of educational trajectories.

Early academic performance constitutes a relevant indicator of the levels of achievement students are likely to attain in subsequent years (Carbonneau et al., 2023; Johnson et al., 2022; Ryu & Lee, 2024; Yeo et al., 2023). This relative stability aligns with findings reported by Hao et al. (2024), which show that students with lower grades tend to remain in declining trajectories, whereas those with adequate performance follow upward patterns. Taken together, these results underscore the relevance of early interventions aimed at modifying low-achievement pathways, given that initial performance reflects not only prior academic competencies but also behavioral and motivational adjustment processes that consolidate over time.

At the individual level, gender shows differentiated associations with both academic outcomes and indicators of emotional adjustment across educational trajectories (Erentaitė et al., 2023; Fréchette-Simard et al., 2023; Oh, 2023). Females tend to display more favorable performance patterns in certain domains while simultaneously exhibiting higher levels of test anxiety during critical transitions, pointing to a potential dissociation between achievement and emotional well-being. In addition, prosocial behaviors and adequate levels of assertiveness are linked to higher academic outcomes (Ma et al., 2026), reflecting the development of socioemotional competencies that facilitate positive peer relationships and promote school engagement (Martinsone et al., 2025; Y. Zhao & Sang, 2025). By contrast, aggressive behaviors are associated with lower academic performance (Tamayo Martínez et al., 2021). Consistent with these patterns, students presenting emotional, behavioral, and attentional problems tend to show lower initial scores and slower growth rates in reading and mathematics (Shi et al., 2023).

Within this framework, self-regulation emerges as a central mechanism in shaping favorable academic trajectories. The capacity for emotional and behavioral self-management is associated with greater goal attainment and, consequently, higher academic achievement (Carpendale et al., 2025; Vasilopoulos & Ellefson, 2021). The findings of this review reinforce previous evidence linking self-regulation to more stable and upward achievement trajectories (Alivernini et al., 2023; Johnson et al., 2022) and further highlight the role of self-regulated learning—planning, monitoring, and goal setting—in achieving better performance on standardized assessments (Bardach et al., 2023). In this process, satisfaction of the psychological needs for autonomy and competence appears to play a key role in the development of self-regulated learning (Lee et al., 2025).

Motivation represents another core dimension underlying educational trajectories, as it enables students to sustain effort, engagement, and performance over time, even in the face of school-related frustration. Evidence from both cross-sectional and longitudinal studies indicates that profiles characterized by high-quality motivation are associated with membership in high-achievement trajectories and groups in mathematics (Santana-Monagas et al., 2025; Lyu & Hu, 2024). Nevertheless, during middle school, a progressive decline in students’ orientation toward learning and personal improvement has been documented (Peixoto et al., 2025), a pattern that extends to later educational stages, including the transition to higher education (Ramos et al., 2021; Scherrer et al., 2024). These motivational changes may be influenced by beliefs developed throughout schooling regarding their own abilities in mathematics and language, which, in turn, affect both motivation and academic performance (Wan et al., 2021). Relatedly, academic goals play a relevant role, as the minimization of effort is associated with declines in academic achievement (Yu et al., 2023).

In parallel, cognitive and metacognitive skills show consistent associations with academic trajectories. The use of metacognitive strategies (Cai et al., 2023), together with specific competencies such as calculation and word-problem-solving skills, is linked to higher performance in mathematics assessments (Mädamürk & Kikas, 2024). More broadly, effortful control is positively associated with long-term academic achievement (Cheng et al., 2024). In contrast, difficulties in working memory are related to negative interactions with teachers and lower arithmetic performance across educational trajectories (Sankalaite et al., 2023). These findings align with prior evidence indicating that working memory, cognitive flexibility, and inhibitory control influence mathematics achievement (Nunes de Santana et al., 2024; Zhu et al., 2025), as well as with studies showing that attentional difficulties affect language development (Santos et al., 2022).

Despite these protective factors, educational trajectories may also be constrained by a range of structural and personal challenges. Students with special educational needs consistently exhibit lower academic achievement (Carroll et al., 2022), particularly those with attention-deficit/hyperactivity disorder (ADHD), who show poorer performance in mathematics and language, with relevant implications for access to postsecondary education (Di Lonardo Burr et al., 2022; Lange-Küttner et al., 2025). Additionally, socioeconomic status—especially in mathematics (Erentaitė et al., 2023)—together with early psychosocial risk, is associated with less favorable achievement trajectories (Farley et al., 2023).

Although the reviewed evidence consistently highlights the longitudinal stability of academic achievement trajectories, this stability should not be interpreted as deterministic. From a behavioral and developmental perspective, trajectories reflect dynamic processes that remain open to change through the interactions between students and their learning environments. Teacher practices, socioemotional support, and structured opportunities for self-regulation can introduce elements of plasticity capable of redirecting initially unfavorable pathways. This tension between continuity and change suggests that academic trajectories are not merely the outcome of early advantages or disadvantages but the expression of cumulative and reciprocal processes that unfold over time.

The school context also plays a critical role in shaping educational trajectories. Support provided by teachers at the emotional, instructional, and classroom management levels fosters student motivation and, consequently, academic achievement (Martin et al., 2024; Ryu & Lee, 2024). Similarly, the quality of the teacher–student relationship shows longitudinal associations with academic and behavioral adjustment, whereas relational conflict is linked to lower academic performance (Magro et al., 2025). Pedagogical practices that emphasize learning and personal progress (mastery-oriented practices) appear particularly relevant for students with low initial performance, promoting progressive gains in achievement (Quintero & Wang, 2023). Consistent with these findings, teacher support emerges as a key factor in sustaining students’ motivation and orientation toward personal improvement (Ramos et al., 2021).

Beyond classroom-level processes, broader contextual conditions also shape academic outcomes. Prior academic achievement is associated with subsequent performance across domains, including the transition from middle school to high school and trajectories of growth in mathematics (Pazeto et al., 2019; Johnson et al., 2022; Yeo et al., 2023; Ryu & Lee, 2024). However, this association is shaped by school context, as high levels of classroom poverty are linked to lower academic achievement and a reduced likelihood of access to higher education (Huang et al., 2025). Moreover, inequalities in student distribution and school location influence academic outcomes (Clements & Sarama, 2025; Ibiapina & Monteiro, 2024; Ramlackhan & Wang, 2024). Importantly, socioeconomic disadvantage and migration-related factors should be understood as analytically distinct, although potentially interacting, dimensions influencing academic trajectories. Within this broader context, migration constitutes an additional challenge, as migrant students face structural and contextual barriers that hinder academic achievement, while also showing significant progress over time (Le Pichon et al., 2023; Ochoa et al., 2024; Lo et al., 2025; S. Y. Kim et al., 2025).

It is also important to note that the included studies were conducted across diverse educational contexts, reflecting the heterogeneity of school systems at the international level. Differences in institutional structures, educational policies, and sociocultural conditions across countries may influence both the configuration of academic trajectories and the way factors associated with academic achievement operate. Accordingly, the findings of this review should be interpreted in light of these contextual differences, as the identified patterns may not manifest uniformly across educational systems.

Finally, the results indicate that factors influencing educational trajectories—typically examined within stable school contexts—may operate differently under unexpected circumstances, such as the COVID-19 pandemic. In this context, a significant decline in academic achievement was observed, particularly in reading and in lower grade levels, with a more pronounced impact among students attending schools with high levels of poverty (Kuhfeld et al., 2023). Nevertheless, the evidence is not entirely homogeneous, as some studies do not report significant losses in specific domains, such as mathematics, suggesting differentiated contextual effects depending on the country, the academic domain, and institutional conditions (Compagnoni et al., 2025).

Overall, the included studies demonstrated adequate methodological quality according to the JBI appraisal, although some limitations were identified. These were mainly related to the reporting of measurement aspects, which may affect the robustness of the findings without compromising the overall consistency of the evidence. These aspects should therefore be considered when interpreting the results.

In summary, this review contributes to a more integrated understanding of academic achievement trajectories by synthesizing longitudinal evidence across multiple ecological levels. By examining stability, change, and the interplay between individual and contextual factors, the findings highlight both the cumulative nature of achievement development and the potential for redirection through targeted interventions. Considering the contextual conditions in which these trajectories develop is therefore essential for informing more relevant educational interventions and for addressing inequalities that influence academic achievement. At the same time, the predominance of individual-level explanatory models identified in the literature underscores the need for a broader incorporation of structural determinants in future research. These considerations frame the scope and interpretation of the present findings and lead to several methodological and conceptual limitations that warrant attention.

### 4.1. Limitations of the Study

Although this review included three widely recognized databases (Web of Science, Scopus, and ERIC), enabling access to high-quality research, this selection necessarily excluded other potentially relevant databases. In addition, grey literature and documents from institutional repositories were not considered, which may have led to the omission of relevant non-peer-reviewed studies.

The search was limited to articles published in English and Spanish, which may have resulted in the exclusion of relevant research in other languages. The temporal delimitation, intended to capture recent studies and avoid overlap with previous reviews, may also have restricted the inclusion of longer-term longitudinal evidence.

This review focused on educational trajectories between primary and secondary education, excluding preschool and higher education. This focus may limit the generalizability of the findings, as factors influencing academic achievement can vary across educational stages.

Taken together, these limitations define the scope of the present synthesis and should be considered when interpreting the findings.

### 4.2. Future Research Directions

Further research should extend the examination of educational trajectories across the educational system using longitudinal designs spanning from early childhood to higher education. This approach may contribute to a more precise understanding of the mechanisms underlying continuity and change in academic achievement.

Greater attention is warranted for specific student populations. Research on students with special educational needs remains essential, particularly given the diversity of profiles and contexts, as well as the role of inclusive practices in shaping equitable educational pathways. A stronger focus on the educational trajectories of migrant students could also clarify the structural, linguistic, and cultural barriers affecting their academic outcomes and access to tertiary education.

Moreover, greater attention to contextual and structural factors is needed, as the existing literature has predominantly focused on individual-level factors, limiting a more comprehensive understanding of the broader influences on academic achievement trajectories.

Systematic reviews focusing on transitions into higher education represent another relevant direction, as these transitions involve selective processes largely based on academic achievement. Such evidence syntheses may help identify the personal, academic, and contextual factors associated with access, persistence, and success in higher education, thereby informing the development of more equitable educational policies and practices.

## 5. Conclusions

This study addressed its objective of examining the personal, family, school, and contextual factors involved in educational trajectories associated with academic achievement among primary and secondary school students. The findings indicate that these trajectories develop progressively over time, beginning early in schooling and tending to exhibit marked stability in later educational stages. This pattern is particularly relevant, as it suggests that academic inequalities may persist in the absence of timely and sustained interventions. From a behavioral and developmental perspective, academic achievement trajectories reflect not only cognitive skills but also processes of self-regulation, motivation, and socioemotional adjustment that interact with family, school, and contextual conditions.

Personal factors such as prior academic performance, self-regulation, academic motivation, and cognitive and relational skills constitute central mechanisms sustaining favorable trajectories or, conversely, giving rise to risk trajectories. At the family and school levels, contexts may enhance resources through appropriate parenting practices and teacher support, or contribute to the cumulative accumulation of disadvantages over time. The findings also indicate that unexpected contextual factors, such as the COVID-19 pandemic, may differentially modify educational trajectories, highlighting the importance of examining academic achievement from a broad and contextualized perspective.

Overall, the findings underscore the importance of integrated educational approaches that extend beyond an exclusive focus on initial academic achievement and coherently articulate the personal, family, and school-related factors shaping educational trajectories. The development of socioemotional competencies, the strengthening of teacher support, and the promotion of supportive school environments, in collaboration with families, emerge as key elements for sustaining favorable academic trajectories and for facilitating change in those characterized by greater difficulties, thereby contributing to more equitable and sustainable educational development.

## Data Availability

No new data were created or analyzed in this study. Data sharing is not applicable.

## Supplementary Materials

The following supporting information can be downloaded at: https://www.mdpi.com/article/10.3390/bs16040608/s1, Table S1. Characteristics and main findings of the included studies.
