# Supplementary material for: Educational Trajectories and Academic Achievement from Primary to Secondary Education: A Systematic Review of Individual, Family, School, and Contextual Factors
Source: Behav Sci (Basel). 2026 Apr 19;16(4):608. doi: 10.3390/bs16040608 (PMC13113718; doi:10.3390/bs16040608)
Supplement: Supplementary file 1 [file behavsci-16-00608-s001.zip › behavsci-4177579-supplementary.pdf]

Table S1. Characteristics and main findings of the included studies

STUDY ID & CONTEXT	STUDY DESIGN	SAMPLE	KEY BACKGROUND VARIABLES	MAIN DOMAIN OF ASSOCIATION	RESULTS
**Alivernini et al. (2023) - Italy**	Observational longitudinal; Latent Growth Models (LGM)	N = 228; age range = 10-16 years; Mage = 12.80 years (SD = 1.74); 56.6% male	Personal: Motivation types (intrinsic, identified, introjected, external), gender, immigrant background. Family: Parents' autonomy support. School/Peers: Teachers' autonomy support, peer acceptance, peer friendship.	Personal Factors: Motivation, Goals & Mindsets	Prior academic achievement (AA) was not associated with initial levels of identified regulation; however, a marginal association was observed between prior AA and subsequent changes in identified regulation over time.
**Bardach et al. (2023) - Germany**	Observational longitudinal; Latent Growth Models (LGM)	N = 1,542; Mage (grade 5) = 11.79 years (SD = 0.71); grades 5-9; 51.75% female	Personal: Self-regulated learning (SRL), externally regulated learning (ERL), gender, immigrant status. Family: Parent autonomy support, parental achievement pressure. School: Teacher autonomy support, teacher achievement pressure, class-level achievement.	Personal Factors: Self-Regulation & Cognitive Functions	Students who show higher levels of self-regulated learning achieve better results on standardized tests both at the beginning of the study and over time; however, they do not necessarily obtain higher school grades.
**Beattie et al. (2025) - Finland**	Observational longitudinal study; Longitudinal Latent Profile Analysis (LPA)	N = 2,567; age range = 12-19 years; grade 6; 55% female, 40% male, 5% other	Personal: Loneliness trajectories, gender, language background. School/Context: Sense of belonging (to school, friends, community).	Personal Factors: Mental Health, Well-Being, & Personality	Differences in GPA were observed across loneliness trajectories, with the "stable high" trajectory exhibiting the highest GPA and the "stable low" trajectory exhibiting the lowest GPA.
**Becker et al. (2023) - Germany**	Observational longitudinal study; Random-Effects Logit Models	N = 5,542; Mage (grade 5) = 11 years; grades 5-9; 52% male	Personal: Educational aspirations (idealistic vs. realistic), immigrant background, gender. Family: Parental educational level (Abitur status). School: School track (Gymnasium vs. non-academic).	Familial Factors: Cultural Capital & Migratory Context	A positive association was observed between school achievement (grades) and both idealistic and realistic educational aspirations. This association strengthened over the course of secondary schooling (grades 5 to 9).
**Bouffard et al. (2023) - Canada**	Observational longitudinal study; Latent Class Growth Analysis (LCGA)	N = 776; Mage (grade 6) = 12.4 years (SD = 0.52); grades 6-11; 52.2% female	Family: Perceived conditional parental regard (parents' affection dependent on academic success). Personal: Anxiety, self-regulation, attitudes toward dropout. School: Teacher-rated motivation.	Familial Factors: Parenting Styles & Parental Support	The trajectory characterized by low and stable perceived conditional parental regard ("low stable") showed higher levels of motivation and self-regulation, as well as less positive attitudes toward school dropout, compared with the other trajectories. Students in the "low stable" group also exhibited marginally higher AA than those in trajectories characterized by higher or increasing levels of conditional parental regard.

**Cabrera-Hernández et al. (2023) - Mexico**	Observational longitudinal study; Interaction-Weighted (IW) estimator	N (ENLACE) = 4,604,135; N (COMIPEMS) = 2,415,382; Mage = 15.17 years; grades 3-6; 50.6% female	School: Full-Time Schools (FTS) program extension (lengthening school day). Personal: Gender. Family: Mother's education level, socioeconomic status (Progresa beneficiary).	School-level Factors: Educational Reforms & Programs	Full-time schools were positively associated with students' attendance at more selective secondary schools. These effects were cumulative and long-term. In addition, increases were observed in entrance examination scores for highly selective upper secondary schools. These effects were observed across students, but were more pronounced among girls.
**Cai et al. (2023) - China**	Observational longitudinal study; Multivariate Latent Growth Modeling (MLGM)	N = 6,776; age range = 12.21-14.22 years; Mage = 13.23 years (SD = 1.06); 52.0% male	Personal: Utility value (intrinsic/extrinsic), metacognitive strategies, gender. School: School ability banding (Band 1, 2, 3).	Personal Factors: Self-Regulation & Cognitive Functions	Increases in AA were positively associated with increases in the use of metacognitive strategies. Higher initial levels of metacognitive strategies were associated with a slower decline in intrinsic utility value (UV-intrinsic). While intrinsic utility value decreased over time, AA increased.
**Carbonneau et al. (2023) - Canada**	Observational longitudinal study, Latent Class Growth Mixture Models (LCGMM)	N = 1,538; grades 1-6; 51.9% female	Personal: School readiness, temperament, sex. Family: Maternal depression, family dysfunction, parenting practices (coercive/overprotective). Context: Socio-familial risk index (prenatal/early childhood).	Familial Factors: Predictors of Early Development	AA followed differentiated trajectories that were associated with socio-familial, parental, and child characteristics. Children exposed to multiple early risk factors tended to follow lower AA trajectories, whereas those in more advantaged environments showed more sustained developmental patterns.
**Carroll et al. (2022) - Ireland**	Observational longitudinal study; Binary Logistic Regression	N (total) = 8,570: N (Wave 1) = 8,570, N (Wave 3) = 6,216; age points = 9, 13, 17-18 years; gender distribution not reported	Personal: Special Educational Needs (SEN) status, self-concept. Family: Conflict with primary caregiver, economic vulnerability. School: Quality of interaction with teachers, learning support provision, DEIS status (disadvantaged school).	Personal Factors: Trajectories of Special Needs	Lower reading achievement at age 9 was associated with a higher likelihood of early school leaving and a lower likelihood of planning to attend higher education. In addition, positive interactions with teachers were associated with a lower likelihood of early school leaving and more favorable post-secondary educational plans.
**Cheng et al. (2024) - United States**	Observational longitudinal study; Latent Growth Curve (LGC) Models	N = 674; age range = 10-23 years; Mage (5 grade) = 10.9 years (SD = 0.51); grade = from 5th grade to 6 years post-high school; 50% male	Personal: Temperament trajectories (Effortful Control [EC], Negative Emotionality [NEM]). Family: Parental monitoring (knowledge of child's activities).	Personal Factors: Mental Health, Well-Being, & Personality	High initial EC predicted higher GPA, test scores, greater course rigor, and higher rates of high school graduation and college attendance. In contrast, higher initial levels of NEM were associated with poorer academic outcomes. Parental monitoring moderated these associations, with higher levels of monitoring buffering the negative impact of NEM and attenuating the associations between EC and academic outcomes.
**Compagnoni et al. (2025) - Switzerland**	Observational longitudinal study; Latent Growth Curve (LGC) Models	N = 1,299; Mage = 11.79 years (SD = 0.48); grade 5; 52% male	Context: COVID-19 school closures (impact of 8-week disruption). Personal: Mathematical self-concept, gender, language spoken at home. Family: Socioeconomic status (books at home).	Contextual & Systemic Factors: Impact of Disruptive Events	The school closures did not result in significant learning loss in mathematics competencies; the intervention cohort even showed marginally better results. Mathematical self-concept declined slightly over the year. Reciprocal associations were observed, with higher and more stable mathematical self-

					concept predicting greater learning gains, and higher achievement predicting higher self-concept.
**Connelly et al. (2024) - United States**	Quasi-experimental longitudinal studies; Fixed-Effects Ordinary Least Squares (OLS) Regression Models	N = 905; age (Wave 1) = 11-17 years; Mage (Wave 1) = 13.31 years (SD = 1.79); 63.36% female	School/Context: Participation in structured vs. unstructured extracurricular activities. Family/Context: Out-of-home placement types (foster care, kin care), caregiver involvement, caregiver education.	Familial Factors: Extracurricular Enrichment	Moderate participation in structured extracurricular activities was associated with increases in both mathematics and reading achievement. In contrast, unstructured activities showed no significant association with AA.
**Di Lonardo-Burr et al. (2022) - Canada/United States**	Observational longitudinal study; Latent Growth Curve (LGC) Models	N = 749 (66.6% ADHD); age range = 7-25 years (Trajectory Focus: 9-17); 79% male	Personal: ADHD symptom trajectories (inattention, hyperactivity-impulsivity), medication status. School: Special education services receipt.	Personal Factors: Trajectories of Special Needs	During secondary school, GPA trajectories differentiated adolescents with ADHD: those who later enrolled in postsecondary education showed GPA improvement, whereas those who did not enroll showed declines. Although symptom severity was lower among those who enrolled, GPA trajectories were more strongly associated with postsecondary enrollment.
**Erentaitė et al. (2023) - Lithuania**	Observational longitudinal study; Growth Mixture Modeling (GMM)	N = 17,685; grades 6-8; 50.9% male	Context: COVID-19 pandemic period. Personal: Gender, special education needs (SEN), prior math achievement. Family: Socioeconomic status (SES).	Contextual & Systemic Factors: Impact of Disruptive Events	Yearly mathematics GPA declined from grades 6 to 8 and was positively associated with national mathematics assessment scores. Girls obtained higher grades across all periods, but not higher national test scores. Students with low socioeconomic status and special educational needs showed lower outcomes in both grades and national assessments.
**Farley et al. (2023) - Chile**	Observational longitudinal study; Linear Mixed Effects Models	N = 9,736; Mage (grade 1) = 6.80 years (SD = 0.46); grades 1-8; 50.9% male	Personal: Psychosocial functioning (PSC-CL), classroom adaptation (TOCA-R), chronic illness. Family: Family psychopathology, social isolation, father absence.	Personal Factors: Predictors of Early Development	AA, operationalized as GPA (in grades 3, 6, and 8), was consistently lower among students who exhibited psychosocial risk in first grade.
**Fréchette-Simard et al. (2023) - Canada**	Observational longitudinal; Latent Growth Models (LGM)	N = 478; Mage (Wave 1) = 12.15 years (SD = 0.43); grades 6-7; 51.6% female	Personal: Test anxiety trajectories, internalizing behaviors, academic self-concept, motivation (task value/expectancy), gender.	Personal Factors: Mental Health, Well-Being, & Personality	Lower initial mathematics achievement was associated with higher levels of test anxiety at the end of elementary school. In contrast, language achievement was not significantly related to test anxiety. AA did not predict changes in test anxiety during the transition to secondary school. Test anxiety was higher among girls than among boys.
**Høstmælingen et al. (2025) - Norway**	Observational longitudinal study; Second-Order Growth Mixture Modeling (SOGMM)	N = 2,147; age range = 9-12 years; grades 4-7; gender not reported	Personal: Behavioral Inhibition System (BIS), Behavioral Activation System (BAS) (Drive, Reward Responsiveness, Fun Seeking).	Personal Factors: Motivation, Goals & Mindsets	Three academic progression trajectories were identified (High/Increasing, Moderate/Stable, Low/Decreasing). Membership in the Low/Decreasing trajectory, reflecting poorer academic progression, was associated with higher avoidance sensitivity (BIS), higher Drive, and male gender.

**Huang et al. (2025) - China**	Quasi-experimental longitudinal studies; Fixed-Effects Ordinary Least Squares (OLS) Regression Models	N = 5,124; grades 7-9; 55.7% aged < 13 years; 51.2% male	School: Classroom poverty rate (peer spillover effects), teacher characteristics (experience, education). Family: Parental employment, health, education. Personal: Cognitive and non-cognitive ability, Hukou status.	School-level Factors: Socioeconomic Stratification and Segregation	Higher classroom poverty rates were associated with lower AP, particularly test scores, as well as with lower non-cognitive skills and reduced rates of subsequent high school and college enrollment. These associations were asymmetric and were more pronounced among poor and middle-income students.
**Hwang et al. (2023) - United States**	Observational longitudinal study; Fixed Effects Models	N = 947,558; grades 3-8; 51.0% male	School: Student-teacher racial/ethnic matching, classroom organization (self-contained vs. departmentalized). Personal: Race/ethnicity, English Learner (ELL) status, special education enrollment. Family: Free/Reduced Lunch eligibility.	School-level Factors: Quality of Teacher-Student Relationship & Instruction	Overall, no positive average association is found between having a teacher of the same race/ethnicity and AA. However, in elementary school and in self-contained classrooms, Black students exhibit small but statistically significant improvements in achievement.
**Johnson et al. (2022) - United States**	Observational longitudinal study; Hierarchical Generalized Linear Models (HGLM)	N math = 363,686; N reading = 363,959; grades 6-8; 51% male	Personal: Race/ethnicity, gender, initial achievement level. School: School poverty concentration (% FRPL), racial composition of school, school mobility (changing schools).	School-level Factors: Socioeconomic Stratification and Segregation	Students followed stable academic trajectories, and middle school test scores strongly predicted high school test scores. Male, Black, and Hispanic students, as well as those attending schools with higher proportions of low-income peers, were more likely to fall off track.
**Kim et al. (2025) - United States**	Observational longitudinal study; Structural equation modeling (SEM)	N = 604; Mage (Wave 1) = 12.38 years (SD = 0.92); 54% female	School: Perceived academic discrimination (from teachers/peers), school engagement. Family: Language brokering (translating for parents), brokering stress, parent-child relationship quality.	Familial Factors: Cultural Capital & Migratory Context	Perceived academic discrimination was indirectly associated with lower educational expectations primarily through high school grades, and secondarily through school engagement. These indirect associations were moderated by language brokering: positive parent-child relationships related to language brokering attenuated the negative associations, whereas language brokering stress was linked to lower grades, reduced school engagement, and lower educational expectations.
**Kuhfeld et al. (2023) - United States**	Observational longitudinal study; NWEA MAP Growth Reading Assessments	N= 5,183,849; grades 3-8; 51.2% male	Context: COVID-19 pandemic timing (Fall 2019 vs. 2020 vs. 2021). Personal: Race/ethnicity, grade level. School: School poverty level (high vs. low poverty schools).	Contextual & Systemic Factors: Socioeconomic Stratification and Segregation	Reading test scores in grades 3-8 were lower in 2021 compared with pre-pandemic levels (2019), with larger declines observed in grades 3-5. In addition, students of color attending high-poverty elementary schools experienced the greatest decreases in reading achievement.
**Lange-Küttner et al. (2025) - Germany**	Observational longitudinal study; Path Modeling with full information maximum likelihood (FIML)	N (Total) = 368; grades 6-9; 75% male<br>Cohort 1: N = 198 (50% ADHD; 50% Control); Mage = 12.50 years; 71.7% male<br>Cohort 2: N = 170 (50% ADHD; 50% Control);	Personal: ADHD diagnosis, gender, migration background. Family: Parents' education (CASMIN index). School: School type/track (secondary education).	Personal Factors: Trajectories of Special Needs	Students with ADHD had consistently lower school grades than peers without ADHD, although the association weakened over secondary education. Achievement gaps were evident only among students without comorbid dyslexia, while parental education, migration background, and gender were also associated with grades, particularly in Cohort 2.

		Mage = 12.83 years; 78.8% male			
**Larsen et al. (2023) - Australia**	Observational longitudinal; Latent Growth Models (LGM)	N = 1,150; Mage (grade 3) = 8.59 years (SD = 0.39); Mage (grade 9) = 14.56 years (SD = 0.43); 53% female	School: School sector (Public, Catholic, Independent). Family: Socioeconomic status (parents' education, occupation, home location advantage). Personal: Prior NAPLAN achievement.	Contextual & Systemic Factors: Institutional Policies & Classification Mechanisms	Socioeconomic status, comprising parental education, parental occupational status, and residential location, was significantly associated with AA. Prior NAPLAN achievement was the strongest predictor of subsequent AA across all assessed domains.
**Le Pichon et al. (2023) - Netherlands**	Observational longitudinal study; First Principal Component (PC1)	N = 125; age range = 6.25-13.50 years; Mage = 8.81-10.52 years; 51% male	Personal: Newcomer/Refugee status, willingness to communicate. School: Social inclusion, peer relationships in multilingual settings.	Contextual & Systemic Factors: Institutional Policies & Classification Mechanisms	Newcomers exhibited naturally steeper growth slopes; this school factor was associated with a catch-up pattern in reading comprehension that was not observed in standardized scores, whereas vocabulary gaps persisted regardless of accommodation. As a result, norm accommodation was associated with persistent academic gaps and differential implications for high-stakes tracking decisions.
**Lee et al. (2025) - South Korea**	Observational longitudinal; Latent Growth Models (LGM)	N=1105; grades 5-9; 62.6% male	Personal: Self-regulated learning (SRL) trajectories. Context: Online learning environment. Personal/School: Satisfaction of basic psychological needs (autonomy, competence, relatedness).	Personal Factors: Self-Regulation & Cognitive Functions	Achievement levels (low/medium/high) moderated SRL trajectories, with intermediate achievers displaying the strongest sustained growth. While autonomy and competence universally predicted initial SRL, relatedness was a significant predictor exclusively for the high-achievement group
**Li et al. (2025) - China**	Observational longitudinal study; Parallel-Process Latent Class Growth Modeling (PP-LCGM)	N = 3,132; Mage age = 9.88 years (SD = 0.72); grades 3-7; 54.1% male	Personal: Internalizing and externalizing symptoms co-development. Family: Maladaptive parenting (psychological control, harsh punishment). School/Peers: Peer victimization (verbal, physical, relational).	Familial Factors: Parenting Styles & Parental Support	Six heterogeneous co-developmental trajectories of AA were identified, including an optimal group with high and stable achievement and low levels of internalizing and externalizing symptoms. Maladaptive parenting and verbal and relational bullying victimization were associated with membership in academically adverse trajectories.
**Lo et al. (2025) - United States**	Observational longitudinal study; Structural Equation Modeling (SEM)	N = 444; age range = 12-15 years; Mage = 13.04 years (SD = 0.73); grades 6-8; 53.8% female	Family: Fathers' and mothers' cultural orientations (Chinese vs. US vs. Bicultural), acculturative stress, supportive parenting behaviors. Context: Parent-adolescent alienation.	Familial Factors: Cultural Capital & Migratory Context	From early to middle adolescence, mothers' bicultural and more U.S.-oriented cultural orientations predicted increases in adolescents' GPA indirectly through lower acculturative stress, higher supportive parenting behaviors, and lower parent-adolescent alienation. Fathers' cultural orientations showed no significant indirect effects on GPA.
**Lyu & Hu (2024) - China**	Observational longitudinal study; Latent Class Growth Modeling (LCGM)	N = 3,772; Mage (Wave 1) = 9.43 years (SD = 0.54); grades 4-6; 52.7% male	Personal: Motivation profiles (combinations of autonomous and controlled motivation), quantity vs. quality of motivation.	Personal Factors: Motivation, Goals & Mindsets	Four math achievement trajectories were identified. Students with a high quality motivation profile characterized by high levels of autonomous motivation and low levels of controlled motivation were overrepresented in

					the most favorable achievement trajectories, whereas the remaining motivation profiles were associated with less favorable math achievement trajectories.
**Ma et al. (2025) - China**	Observational longitudinal study; Parallel-Process Latent Class Growth Modeling (PP-LCGM)	N = 1,012; Mage = 9.49 years (SD = 0.70); grades 3-6; 51.2% female	Personal: Social assertiveness, prosociality. Family: Parental education. Context: Sibling status (only child vs. siblings).	Personal Factors: Class Ecology & Peer Dynamics	Students in trajectories with higher prosociality showed higher AP, whereas those in low prosociality and low assertiveness trajectories showed lower performance. AP at the initial assessment was positively associated with prosociality and assertiveness across all waves.
**Mädamürk & Kikas (2024) - Estonia**	Observational longitudinal study; Latent Transition Analysis (LTA)	N = 1,023; age range = 9-16 years; grades 1-9; 52.1% male	Personal: Calculation skills, word-problem solving skills. Family: Parental education. School: Educational path (high school vs. vocational vs. dropout).	Personal Factors: Self-Regulation & Cognitive Functions	The study shows that students who consistently remained in the high-skills profile from grades 1 to 9 achieved the highest scores on the math examination (advanced and intermediate levels) and on the Estonian language examination, assessed as later outcomes in grade 12.
**Magro et al. (2025) - United States**	Observational longitudinal study; Latent Curve Model with Structured Residuals (LCM-SR)	N = 1,041; grades K-6; 51.7% female	School: Teacher-Student Relationship quality (Closeness and Conflict). Personal: Social competence, externalizing/internalizing behaviors.	School-level Factors: Quality of Teacher-Student Relationship & Instruction	Within-person analyses indicated that teacher-student conflict was bidirectionally associated with externalizing symptoms and predicted lower subsequent AA, whereas teacher-student closeness showed bidirectional associations with AA. Associations involving conflict were stronger than those involving closeness.
**Martin et al. (2024) - Australia**	Observational longitudinal study; Confirmatory Factor Analysis (CFA)	N = 19,465; grades 6-7; 51% male	School: Perceived Teaching Support (emotional, instructional). Personal: Motivation (valuing, growth goals), engagement, perseverance. Context: Transition from primary to high school.	School-level Factors: Quality of Teacher-Student Relationship & Instruction	Students' motivation in grade 7 significantly predicted their AA during the transition to secondary school. Perceived teacher support positively predicted students' motivation (valuing, growth goals, and aspirations). In turn, growth goals predicted student engagement and formed part of indirect associations linking teacher support to engagement across the secondary school transition.
**Nie et al. (2025) - China**	Observational longitudinal study; Latent Class Growth Modeling (LCGM)	N = 4,294; Mage = 14.22 years (SD = 1.56); grades 7-12; 51.4% male	Personal: Psychological Suzhi (cognitive, individuality, adaptability qualities), mental health (depression, anxiety). Family: Parenting styles. School: Peer victimization.	Personal Factors: Mental Health, Well-Being, & Personality	Adolescents belonging to higher Psychological Suzhi trajectories (stable or decreasing from high levels) showed higher academic achievement three years later compared with those in the low and decreasing trajectory. Psychological Suzhi declined overall during secondary school, with four distinct developmental trajectories identified.
**Ochoa et al. (2024) - United States**	Observational longitudinal study; Mixed-Effects/Multilevel Modeling	N = 30,273; grades K-12; 51.1% male	School: Language support program availability (compliance with Lau v. Nichols). Personal: English Learner (EL) status (labeled vs. reclassified vs. never EL), IEP status.	School-level Factors: Institutional Policies & Classification Mechanisms	Students identified as English Learners (EL) showed positive and statistically significant gains in mean English Language Arts (ELA) scores over time when attending schools with a language support program. However, EL students continued to obtain lower ELA scores than peers with higher English proficiency.

**Oh (2023) - South Korea**	Observational longitudinal study; Multilevel Latent Growth Curve Model (MLGCM)	N = 4,051; grades 7-9; 51.4% male	Personal: L2 (Second Language) self-efficacy, L2 interest, L2 proficiency. School: L2 classroom engagement (attitude, comprehension), Teacher efficacy. Family: Private tutoring expenditure, family income.	Familial Factors: Socioeconomic Stratification and Segregation	Regarding L2 achievement trajectories, the results showed that L2 classroom comprehension and higher initial L2 proficiency had positive effects on the initial level of L2 achievement but were not associated with its growth rate. In contrast, being female was positively associated with both the initial level and the growth rate of L2 achievement.
**Peixoto et al. (2025) - Portugal**	Observational longitudinal study; Latent Class Growth Modeling (LCGM)	N = 1,574; age range = 10-14 years; Mage = 11.44 years (SD = 1.26); grades 5-7; 50% female	Personal: Achievement goals (task, self-enhancing, self-defeating, avoidance). School: Grade retention (repeating a year). Family: Mother's education.	Personal Factors: Motivation, Goals & Mindsets	AA showed a general decline over time. Declines in task and self-enhancing goals were positively associated with achievement, whereas increases in avoidance goals were negatively associated with performance. Younger students exhibited steeper declines, and grade retention was linked to lower initial achievement but flatter subsequent trajectories.
**Qi et al. (2025) - China**	Observational longitudinal study; Latent Class Growth Analysis (LCGA)	Study 1 (Urban): N = 748; Mage = 10.23 (SD = 0.30); Study 2 (Rural): N = 3,258; Mage = 10.34 (SD = 0.37); Both: grades 4-6; 51% male	Personal: Intelligence mindsets (Growth vs. Fixed vs. Mixed profiles), IQ. Family: Socioeconomic status (parental education/income).	Personal Factors: Motivation, Goals & Mindsets	Students in the Growth profile (predominantly growth mindsets) showed the largest improvements in AA two years later, compared with the Both-High profile (simultaneously high growth and fixed mindsets), which exhibited the least favorable academic outcomes. The Fixed profile (predominantly fixed mindsets) did not show the lowest AA.
**Quintero & Wang (2023) - United States**	Observational longitudinal study; Confirmatory Factor Analysis (CFA)	N = 784; age range (grade 4) = 5.25-8.25 years; Mage (grade 4) = 6.57 years (SD = 0.40); grades 4-9; 53% male	School: Classroom goal structures (Mastery, Performance-Approach, Performance-Avoidance). Family: Socioeconomic status (education, occupation, free lunch eligibility).	Personal Factors: Class Ecology & Peer Dynamics	Higher perceived mastery classroom goal structure was associated with faster growth in AA among academically at-risk students, regardless of family socioeconomic status. In contrast, higher perceived performance classroom goal structures were associated with lower AA growth and with an attenuation of the academic advantage associated with higher socioeconomic status.
**Ramlackhan & Wang (2024) - United States**	Observational longitudinal study; Growth Mixture Modeling (GMM)	N = 1,887 urban public-school districts; grades 3-8	Context: Neighborhood income segregation, School income segregation. School: Title I status, school racial composition. Personal: Race/ethnicity.	School-level Factors: Socioeconomic Stratification and Segregation	AA trajectories in English Language Arts and mathematics varied by districts' racial and socioeconomic composition. In mathematics, districts with higher proportions of Hispanic students clustered in lower-achievement trajectories, whereas those with higher proportions of White students clustered in higher-achievement trajectories.
**Raufelder et al. (2022) - Finland**	Observational longitudinal study; Latent Transition Analysis (LTA)	N = 1,073; Mage = 12.33 years (SD = 0.37); grades 6-9; 54.5% female	Personal: Motivational profiles (Expectancy-Value Theory components: utility, attainment, cost). Family: Parental education level.	Personal Factors: Motivation, Goals & Mindsets	Students in the "struggling ambitious" profile showed the highest levels of AA in mathematics and literacy (reading and spelling skills), outperforming even the positively engaged profile.
**Ryu & Lee (2024) - South Korea**	Observational longitudinal; Latent Growth Models (LGM)	N = 3,530 (Total Cohort); N = 2,740	Personal: Working memory (perceived and performance-based).	School-level Factors: Socioeconomic	English achievement increased steadily from grade 4 to 9. Students with higher initial achievement maintained a sustained advantage over time, whereas students with

		(Analytical Sample); grades 4-9; 50.8% male	School: Teacher-Student Relationship (closeness, conflict).	Stratification and Segregation	lower initial achievement also improved, but the gap between groups did not narrow; by grade 9, the lower-achieving group did not reach the initial level of the higher-achieving group.
**Sankalaite et al. (2023) - Belgium**	Observational longitudinal study; Cross-Lagged Panel Design (CLPD) with Structural Equation Modeling (SEM)	N = 105; grades 1-6; age range = 6-12 years; Mage = 9.13 years (SD = 1.78); 51.43% male	Personal: Working memory. School: Teacher-Student Relationship quality. Family: Parent-rated executive functions.	Personal Factors: Self-Regulation & Cognitive Functions	Arithmetic performance showed bidirectional associations with perceived working memory problems. Higher teacher-student relationship closeness was associated with fewer subsequent working memory problems, whereas higher perceived working memory problems were associated with greater teacher-student relationship conflict.
**Shi et al. (2023) - United States**	Observational longitudinal study; Growth Mixture Modeling (GMM)	N = 784; Mage (grade 1) = 6.57 years; grades 1-9; 53% male	Personal: Dysregulation Profile (DP) (affective, behavioral, cognitive dysregulation). Family: Socioeconomic adversity (cumulative risk). School: Grade retention.	Personal Factors: Mental Health, Well-Being, & Personality	Two compensatory co-developed trajectories of reading and math performance were identified, and early childhood dysregulation was not associated with trajectory class membership. However, higher levels of dysregulation were associated with lower initial reading and math performance and with slower growth in both domains within trajectories.
**Tseliou et al. (2024) - United Kingdom**	Observational longitudinal study; Latent Class Growth Analysis (LCGA)	N = 27,085; grades 6-11; gender distribution not reported	School: School exclusions (suspensions/expulsions trajectories). Personal: Special Educational Needs (SEN) type. Family: Free School Meal (FSM) eligibility history.	School-level Factors: Institutional Policies & Classification Mechanisms	AP was measured as the attainment of the GCSE. Students belonging to exclusion trajectories, including low-level and high-exclusion trajectories, were less likely to achieve this outcome than those with no exclusions. Associations with attainment were stronger for exclusion frequency than for exclusion timing.
**Wang & Tang (2024) - China**	Observational longitudinal study; Latent Growth Curve Models (LGC)	N = 645; age range = 10.50-18.47 years; grades 4-9; 50.39% female	Family: Parental involvement dimensions (Educational expectations, Homework supervision, Discussion about school, TV rules, Tutoring support). Personal: Gender.	Familial Factors: Parenting Styles & Parental Support	Mathematical performance showed a positive linear growth trajectory over eight years. Parental educational expectations were positively associated with both the initial level and growth rate of mathematical performance. Other forms of parental involvement, including homework supervision and parent-child discussion, were associated with the initial level of mathematical performance, with these associations varying by gender. Girls showed higher mathematical performance in later waves, and more dimensions of parental involvement were associated with their initial performance.
**Wei et al. (2023) - China**	Observational longitudinal study; Parallel Latent Growth Curve Models (PLGCM) and Random-Intercept	Middle School Cohort: N = 880; grades 7-9; Mage = 13.33 (SD = 0.64); 54.5% male High School Cohort: N = 646; grades 10-12;	School/Peers: Popularity (perceived popular), Peer acceptance (sociometric preference). Personal: Aggression.	Personal Factors: Class Ecology & Peer Dynamics	A reciprocal association was observed between popularity and AA in both lower and upper secondary schools. In contrast, the association between peer acceptance and AA was observed only in upper secondary school, and changes in peer acceptance were not associated with subsequent changes in AA.

	Cross-Lagged Panel Models (RI-CLPM)	Mage = 16.76 (SD = 0.75); 47.1% male			
**Wu & Becker (2023) - Germany**	Observational longitudinal study; Confirmatory Factor Analysis (CFA)	N = 1,841; Mage (grade 6) = 12.20 years (SD = 0.81); grades 6-10; 50.4% female	School: School tracking (Academic vs. Non-academic track), School-level achievement composition. Personal: Life satisfaction, school satisfaction trajectories.	School-level Factors: Institutional Policies & Classification Mechanisms	School-level AA composition, defined as the average academic achievement of students within a school, was associated with longitudinal changes in life satisfaction, with higher-achieving school contexts linked to steeper declines over time. This academic composition was not significantly associated with changes in school satisfaction.
**Yeo et al. (2023) - South Korea**	Observational longitudinal study; Growth Mixture Modeling (GMM)	N = 3,089; Mage (grade 5) = 10.47 years (SD = 0.58); grades 5-9; 49.6% male	School: Instructional styles (Direct instruction vs. Dialogic instruction). Personal: Initial math achievement level (low/average/high). Family: Multicultural family status.	School-level Factors: Quality of Teacher-Student Relationship & Instruction	AP, measured by German school grades, showed a longitudinal decline from grades 5 to 9. Reciprocal associations with classroom management were observed at mid-adolescence, while perceived goal clarity was positively associated with grades in later grades. Persistent differences in AP by gender and school track were observed, without evidence of differential growth trajectories.
**Yeung & Igarashi (2025) - United States**	Observational longitudinal study; Parallel-Process Latent Growth Curve Modeling (PP-LGCM)	N = 500; Mage = 13.40 years (SD = 0.60); 52.8% female	Personal: Educational expectations, Science performance trajectories. Family: Parental SES, family composition (two-parent vs. other). Context: Ethnicity.	Personal Factors: Trajectories of Special Needs	AP in mathematics and physics, assessed across 12 semesters, was higher for non-music students than for music students in most semesters. Music training, parental education, and out-of-school academic engagement were associated with the level of AP, but not with longitudinal change in performance.
**Yu et al. (2023) - Finland**	Observational longitudinal; Latent Growth Models (LGM)	N = 2,680; grades 4-8; 53.9% male	Personal: Achievement goal orientations (Mastery, Outcome, Performance-Approach, Performance-Avoidance, Work-Avoidance). Context: Well-being (burnout, life satisfaction).	Personal Factors: Motivation, Goals & Mindsets	Growth trajectories of mathematics achievement during grades 4 to 6 were associated with subsequent mathematics achievement in grade 8.
**Zhang et al. (2024) - China**	Observational longitudinal study; Compositional Data Analysis (CoDA)	N = 3,116; grades 7-9; 52% male	Personal: Internet addiction, depressive symptoms, Positive Youth Development (PYD). Family: Family function, family structure (intact/non-intact). School: School grade level.	Personal Factors: Self-Regulation & Cognitive Functions	Science performance increased from grades 7 to 9, whereas educational expectations showed a slight decline. Initial educational expectations were positively associated with both the initial level and growth of science performance, which in turn mediated the association between educational expectations and STEM degree attainment in adulthood.
**Zhou & Hawrot (2025) - Germany**	Observational longitudinal study; Latent Growth Models (LGM) and Cross-Lagged Panel Models (CLPM)	N = 1,072; age range = 13-16 years; grades 6-9; 62% female	School: Perceived instructional quality (classroom management, support, cognitive activation). Personal: Intrinsic reading motivation.	School-level Factors: Quality of Teacher-Student Relationship & Instruction	Outcome goals predicted higher GPA, while work-avoidance goals related to lower GPA; mastery goals were linked to subjective well-being rather than GPA.

**Zhou & Lian (2024) - China**	Observational longitudinal study; ANOVA with Autoregressive Model (AR)	N = 1,301; Mage = 12.46 years (SD = 0.63); 51.2% male; 47.7% female; 1.1% gender not reported	School/Activity: Music training (participation in school music programs). Personal: Interest in music. Family: Parents' education.	Familial Factors: Extracurricular Enrichment	Better AP, measured only once at the initial assessment, was associated with a lower likelihood of belonging to the trajectory characterized by initially high levels of internet addiction and depressive symptoms that increased jointly over time.

Note. AA = Academic Achievement; ADHD = Attention-Deficit/Hyperactivity Disorder, AP = Academic Performance; EC = Effortful Control; GPA = Grade Point Average; NEM = Negative Emotionality; SRL = Self-Regulated Learning
